# Retraction: Autoantibodies in renal diseases – clinical significance and recent developments in serological detection

**DOI:** 10.3389/fimmu.2024.1540239

**Published:** 2024-12-16

**Authors:** Frontiers Editorial Office

“Following publication, concerns were raised regarding the integrity of the images in this article. An investigation was conducted in accordance with Frontiers’ policies.

It was found that the concerns were valid and that the article does not meet the standards of editorial and scientific soundness for Frontiers in Immunology; therefore, the article has been retracted.

The retraction was approved by the Chief Editors for Frontiers in Immunology and the Chief Executive Editor of Frontiers. The authors do not approve of the retraction.”

